# Circadian control of stress granules by oscillating EIF2α

**DOI:** 10.1038/s41419-019-1471-y

**Published:** 2019-03-04

**Authors:** Ruiqi Wang, Xin Jiang, Puhua Bao, Meiling Qin, Jin Xu

**Affiliations:** 1 Institute of Neuroscience, State Key Laboratory of Neuroscience, Key Laboratory of Primate Neurobiology, Shanghai Institutes for Biological Sciences, Chinese Academy of Sciences, 320 Yue Yang Road, Shanghai, 200031 China; 2 University of Chinese Academy of Sciences, Shanghai, 200031 China

## Abstract

Stress granule formation is important for stress response in normal cells and could lead to chemotherapy resistance in cancer cells. Aberrant stress granule dynamics are also known to disrupt proteostasis, affect RNA metabolism, and contribute to neuronal cell death. Meanwhile, circadian abnormality is an aging-related risk factor for cancer and neurodegeneration. Whether stress granule dynamics are circadian regulated is entirely unknown. Here we show that the formation of stress granules varied by zeitgeber time in mouse liver. Moreover, altering circadian regulation by silencing the core circadian gene *Bmal1* in a cell line expressing an endogenous GFP-tagged G3BP1 significantly increased stress granule dynamics, while the overexpression of *Bmal1* decreased them. Surprisingly, increased stress granule dynamics and formation by transient decrease of BMAL1 coincided with increased resistance to stress-induced cell death. The circadian regulation of stress granules was mediated by oscillating eIF2α expression. At zeitgeber time when BMAL1 and eIF2α were at nadir, reduction of unphosphorylated eIF2α could significantly alter the ratio of phosphorylated/total eIF2α and quickly lead to increased formation of stress granules. Therefore, diurnal oscillating eIF2α connects the circadian cue to a cellular stress response mechanism that is vital for both neurodegeneration and cancer.

## Introduction

The ability of cells to cope with environmental and cellular stress is vital for their flourishment and survival. Abnormal stress response is known to contribute to aging process and aging-related diseases such as cancer and neurodegenerative diseases^1,2^. By developing various stress response and anti-apoptotic mechanisms, cancer cells can proliferate in hostile microenvironment, such as hypoxia, or even chemotherapy drugs^3^. On the other hand, the inability of neurons to resist increased production of reactive oxygen species and endoplasmic reticulum (ER) stress either during normal aging or under pathogenic conditions will lead to neurodegenerative diseases^4–6^.

One of the cellular stress responses that have been intimately linked to stress resistance in cancer cells and the development of some neurodegenerative diseases is the formation of stress granules^3,7–9^. Stress granules are membrane-less cytoplasmic structures formed when translation initiation is inhibited during strong stress responses or viral infection^10,11^. They are composed of abundant messenger RNAs (mRNAs) stalled in translation initiation, RNA-binding proteins, and ribonucleoproteins. The formation of stress granules and the arrest of canonical translation could serve as a protective mechanism when the cellular resources are limited during stress^10^. While the translation of most constitutive proteins is suppressed, stress-induced mRNAs could be preferentially translated^12^. In tumor cells, stress granule induction promotes resistance to apoptosis in chemotherapy^3^. In neurons, the abnormal regulation of stress granules contributes to neurodegeneration.

Stress granules are dynamic structures characterized by constant exchange of protein components. The exchange rates of those components are different, with the proteins at the dense cores less dynamic^13^, and are affected by the interaction and local concentration of stress granule proteins^14^. Interestingly, a sizable portion of stress granule components are related to the pathogenesis of cancer and/or neurodegenerative diseases, particularly amyotrophic lateral sclerosis (ALS) and frontotemporal dementia (FTD)^13^. Proteins including FUS, TAF15, EWSR1, TDP43, TIA-1, VCP, and Ataxin-2 are not only genetically and/or pathologically related to ALS and FTD, but also involved in cancer development^15–27^. Furthermore, the most common cause of familial ALS and FTD, arginine-rich dipeptide repeats derived from C9orf72 hexanucleotide expansion repeats, could interact with stress granule components, affect the stress granule dynamics, and disrupt nucleocytoplasmic transport^28–31^.

Circadian rhythms are physiological and behavioral changes following an ~24 h cycle. The diurnal changes are governed by a molecular circadian clock, featuring two main feedback transcriptional–translational regulatory loops to direct the oscillating expression of target genes in an organ-specific manner^32,33^. The core circadian proteins BMAL1 and CLOCK transcriptionally activate *Per*, *Cry*, and *Nr1d1/2*, whose protein products, when accumulated, could negatively regulate the expression or transcriptional activity of BMAL1^34,35^. The oscillating expression of these core circadian genes  control the circadian activities. With aging, the circadian control is gradually weakened. There is a clear association between circadian dysregulation and various neurodegenerative diseases^36,37^. Sleep disturbance is frequently seen in patients with Alzheimer’s disease (AD) and Parkinson’s disease (PD)^37–39^, and abnormal circadian behaviors are detected in animal models of AD or PD before the onset of disease pathology^40,41^. In our recent study, we have observed sleep and circadian abnormalities before cognitive deficits in a FUS knock-in rodent model^42^. Therefore, circadian dysregulation could be a risk factor for neurodegenerative diseases. Whether circadian abnormalities may affect cancer risk is uncertain^43^, but DNA excision repair activity appears to be circadian regulated with peak activity at evening in mice^44^. In addition, chronotherapy in cancer has been practiced even though the underlying mechanisms are not entirely clear and the results were mixed^43,45^.

The generation of protective antioxidative enzymes, such as superoxide dismutase, shows diurnal rhythm^46,47^, and this is part of cellular defense system against weak stress insults from reactive oxygen species, the byproducts of normal cellular metabolism. Under ER stress or exogenous stress insults, stress granules will quickly appear. Whether the formation of stress granules could be regulated by circadian cues is entirely unknown. Even if there is such a regulation, as stress granule formation is a fast response to external stress, how would that be regulated by slow circadian signals? In this study, we have provided in vitro and in vivo evidence to demonstrate that stress granule formation is affected by circadian gene expression, and this regulation is due to the oscillation of total eIF2α. We have also unveiled a surprising result showing reduced expression of BMAL1 could protect cells against arsenite stress while promoting stress granule formation. Therefore, our results have suggested an efficient diurnal cellular protective mechanism to guard against increased stress in the active phase, and implied that circadian dysregulation may promote neurodegeneration due to aberrant stress response. In addition, our observations may provide a cellular mechanism to better understand cancer chronotherapy.

## Results

**Fig. 1:**
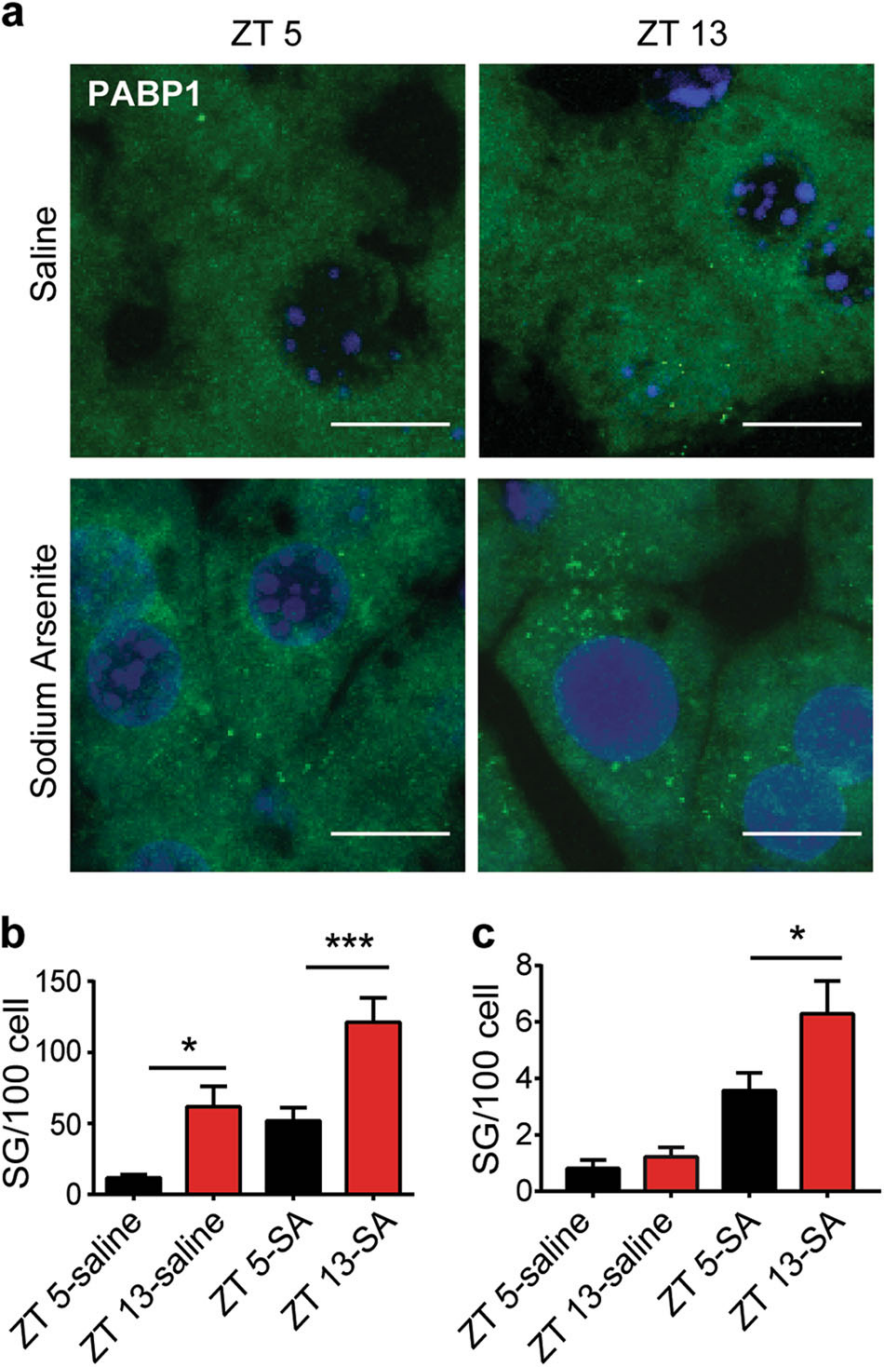
Stress granules in mouse liver at zeitgeber time (ZT) 5 and ZT 13. **a** The liver tissues from the wild-type (WT) mice harvested at ZT 5 and ZT 13 were labeled with anti-PABP1 (stress granule marker) antibody and revealed by immunofluorescence. Mice were intraperitoneally injected with saline or sodium arsenite (SA, 10 mg/kg) for 1 h before killing. Representative micrographs showing the presence of PABP1-positive stress granules (Scale bar = 10 μm). **b**, **c** Quantification of all visible PABP1 puncta (**b**) or large size puncta (>0.5 μm, **c**) respectively (*n* = 3 mice per group with 950–1000 cells examined per mouse. mean ± S.E.M.; two-way analysis of variance (ANOVA) with Tukey’s multiple comparison *P ≤ 0.05, ***P ≤ 0.001)

To examine whether stress granule formation could be affected by the different time of the day in vivo, we intraperitoneally injected the mice with saline (control) or oxidative stressor sodium arsenite and harvested the liver tissues from circadian entrained mice at zeitgeber time 5 and 13 (ZT 5 and ZT 13). The differential expression of core circadian protein BMAL1 was validated (Supplementary Fig. 1a). The liver tissues were selected because liver is an organ with robust circadian-regulated gene expression^48^. Using stress granule markers PABP1 (Fig. 1a) and YB1 (Supplementary Fig. 1b), we have detected higher spontaneous and stress-induced stress granule formation at ZT 13 (Fig. 1a, b, Supplementary Fig. 1b, c). It is worth noting that although the spontaneously occurred stress granules are visible at ZT 13, they were much smaller than the ones formed in sodium arsenite-treated mice. This result suggested that the stress granules could be affected by circadian cues and are more easily formed at night in mice.

**Fig. 2:**
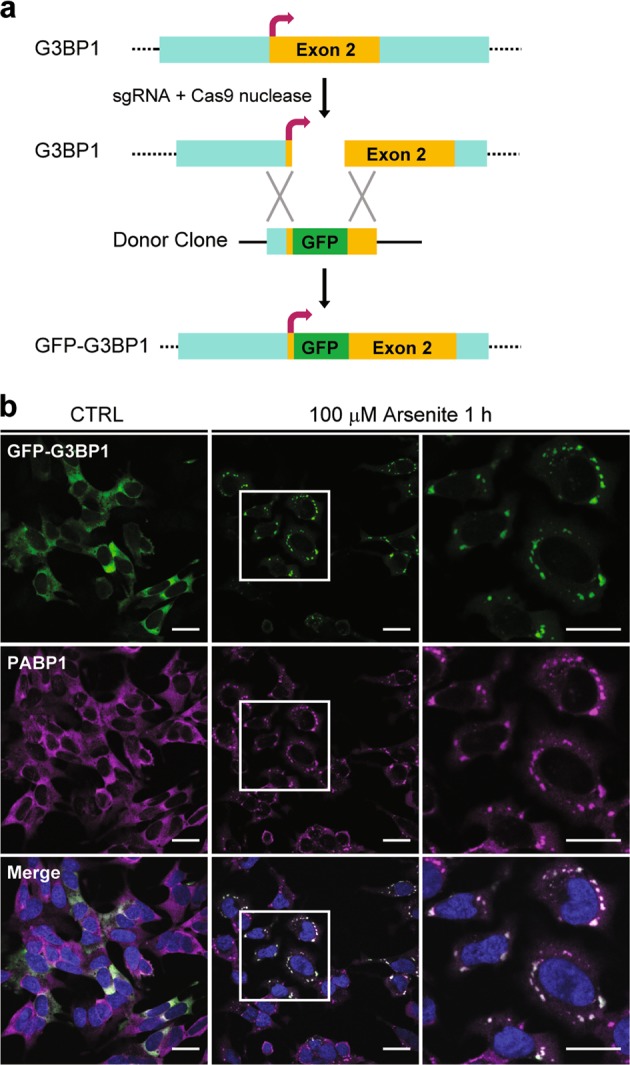
Generation of GFP-G3BP1 knock-in (KI) cell line. **a** Schematic representation of the strategy of tagging green fluorescent protein (GFP) at the N terminus of human *G3BP1* using CRISPR/Cas9 (clustered regularly interspaced short palindromic repeats/CRISPR-associated protein 9). **b** Immunofluorescence confocal microscopy showing the co-localization of endogenous SG marker PABP1 with knock-in GFP-G3BP1. Stress granules were induced with 100 μM sodium arsenite for 1 h. The square areas in the middle panels were enlarged and shown in the right. Scale bar = 20 μm

We then aimed to evaluate the circadian regulation on stress granule formation and dynamics. Overexpression of green fluorescent protein (GFP)-tagged stress granule marker G3BP1 in cell lines is a commonly adopted approach to study stress granule formation. However, we reasoned that exogenous expression of a key protein involved in stress granule assembly^49^ might not be the best approach to study the internal circadian regulation of stress granule formation. Therefore, we used CRISPR/Cas9 (clustered regularly interspaced short palindromic repeats/CRISPR-associated protein 9) gene editing technique to insert a GFP tag in frame to the N terminus of the G3BP1 gene in the human neuroblastoma cell line SH-SY5Y (Fig. 2a, Supplementary Fig. 2a, b). Using this approach, we would retain the normal transcriptional and post-transcriptional regulation of the endogenous G3BP1. SH-SY5Y cells also demonstrated features of oscillating circadian gene expression (Supplementary Fig. 2c, d). The GFP-G3BP1 KI cells expressed G3BP1 in a cytoplasmic diffuse pattern and formed granules that colocalized with stress granule marker PABP1 upon stress (Fig. 2b), indicating the tagged G3BP1 behaved as expected during stress.

**Fig. 3:**
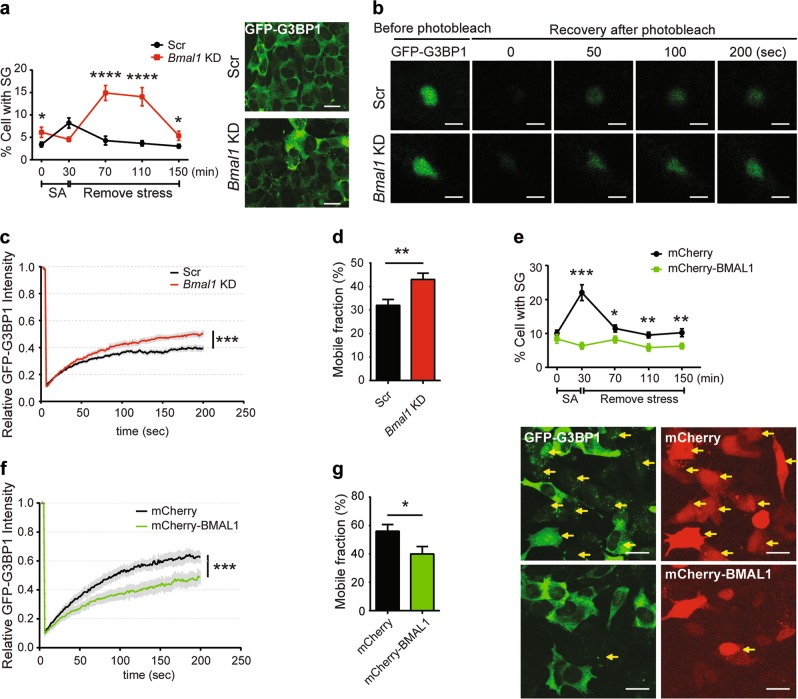
Altered BMAL1 expression affects stress granule (SG) formation and dynamics. **a** *Bmal1* silencing caused increased stress granule formation in GFP-G3BP1 knock-in (KI) cells exposed to sodium arsenite (SA) stress. Cells transfected with the scrambled or *Bmal1* small interfering RNA (siRNA) were treated with 50 μM of SA for 0 or 30 min. After stress was removed, cells were kept in fresh medium for additional 40, 80, or 120 min before fixation for immunofluorescence microscopy analysis. The percentage of cells with stress granules under indicated condition was quantified (mean ± S.E.M.; *n* = 3 independent experiments, with 30 fields for each time point, at least 50 cells per field. *P ≤ 0.05, ****P ≤ 0.0001 by unpaired Student’s-*t*-test, Scale bar = 20 μm). Representative images show the SG formation 80 min after removing stress. **b**–**d** GFP-G3BP1 KI cells were transfected with Cy3-labeled scrambled siRNA (Scr) or *Bmal1* siRNA and subsequently treated with 50 μM SA for 30 min. After stress was removed, green fluorescent protein (GFP)-positive stress granules in CY3-positive cells were analyzed by fluorescence recovery after photobleaching (FRAP). **b** Representative images show the stress granules before and after photobleaching at different time. Scale bar = 2 μm. **c** Signal intensity of GFP fluorescence of stress granule FRAP in Cy3-positive cells. The average fluorescence before photobleaching was designated as 1. **d** Mobile fraction calculated from the FRAP analysis in **c** (mean ± S.E.M.; *n* = 14-15 cells per sample, **P ≤ 0.01, ***P ≤ 0.001 by unpaired Student’s-*t*-test). **e** Overexpression of BMAL1 suppressed stress granule formation. GFP-G3BP1 KI cells expressing mCherry or mCherry-BMAL1 were stress shocked with 20 μM SA and fixed at indicated time for stress granule imaging and quantification (mean ± S.E.M.; *n* = 3 independent experiments, with 30 fields for each time point, at least 10–15 cells per field. *P ≤ 0.05, **P ≤ 0.01 ***P ≤ 0.001 by unpaired Student’s-*t*-test. Scale bar = 20 μm). Representative images show SG formation in cells treated with SA for 30 min. Yellow arrows indicate the SG status of representative mCherry-positive cells included in the quantification. **f** The signal intensity of GFP fluorescence of stress granule FRAP in mCherry-positive cells. **g** Mobile fraction calculated from the FRAP analysis in **f** (mean ± S.E.M.; *n* = 14–15 cells per sample, *P ≤ 0.05, **P ≤ 0.01, ***P ≤ 0.001 by unpaired Student’s-*t*-test)

Bmal1 is a potent circadian regulator and single knockout (KO) of *Bmal1* could cause arrhythmicity^50^. Furthermore, BMAL1 was expressed at low level at ZT 13 in mouse liver when stress granules increased (Fig. 1). Therefore, we chose to silence the expression of *Bmal1* as a way to change circadian input and then evaluated stress granule formation. There was a slight increase of basal stress granule formed upon *Bmal1* silencing (Fig. 3a). Upon transient oxidative stress shock with sodium arsenite for 30 min, the cells with lower BMAL1 expression showed a delayed but significant increased number of cells with stress granules (Fig. 3a). This observation was consistent with the higher number of stress granules formed in mouse liver at ZT 13 (Fig. 1). Next, we examined the change of stress granule dynamic using fluorescence recovery after photobleaching (FRAP) (Fig. 3b). Silencing of BMAL1 led to a statistically significant increase of stress granule dynamic (Fig. 3c) and mobile fraction (Fig. 3d). Interestingly, when we examined the stress granule dynamics in mouse embryonic fibroblasts (MEFs) derived from BMAL1 KO mice, we observed minor decrease of stress granule dynamics and no difference in stress granule formation when compared to the wild-type (WT) MEF (Supplementary Fig. 3a, b), suggesting that the BMAL1 KO cells have different stress response mechanism (see Discussion).

To further validate the effects of BMAL1 on stress granule formation, we overexpressed mCherry-tagged BMAL1 in GFP-G3BP1 knock-in (KI) cells, and quantified the cells with stress granules. In contrast to cells with silenced BMAL1 expression, overexpression of BMAL1 suppressed the formation of stress granules upon stress (Fig. 3e). Furthermore, the stress granule dynamic was also significantly reduced (Fig. 3f), accompanied by decreased mobile fraction (Fig. 3g). To confirm these observations, we examined the regulation of stress granule dynamics in MEF cells derived from NR1D1/RevErbα-deficient mouse embryos. NR1D1/RevErbα is a negative regulator of BMAL1 expression^51^. Depletion of NR1D1/RevErbα led to the reduction of stress granule dynamics and mobile fraction (Supplementary Fig. 3c–e). Taken together, our results showed that the altered expression of core circadian regulators could affect stress granule formation and dynamics.

**Fig. 4:**
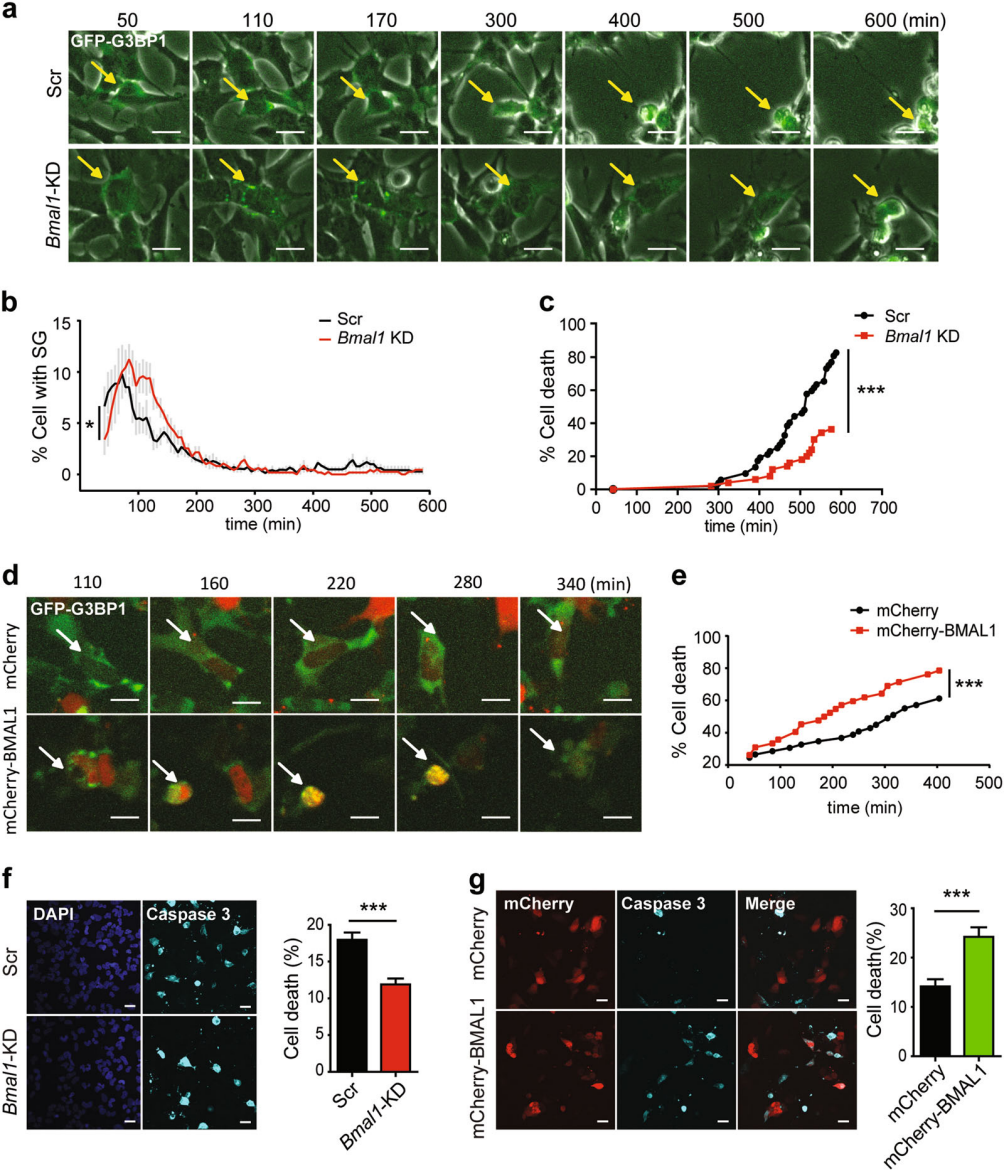
Reduced expression of BMAL1 protects against stress-induced cell apoptosis. **a** Live-cell imaging of *Bmal1*-silenced GFP-G3BP1 knock-in (KI) cells exposed to 20 μM of sodium arsenite for up to 600 min (see Movie S1, 2). The same stress granule-generating cell in each group (Scr or *Bmal1* knockdown (KD)) at different time was indicated by an arrow. Note the different timing of the apoptotic morphology (rounded cell shape and detachment from the dish) in cells transfected with Scr or *Bmal1* small interfering RNA (siRNA). **b** Quantification of the percentage of cells with stress granules (mean ± S.E.M.; *n* = 500–600 cells per sample, *P ≤ 0.05 by two-way analysis of variance (ANOVA). Scale bar = 20 μm). **c** The death ratio of stress granule-positive cells (*n* = 50–100 cells per group, ***P ≤ 0.0001 by log-rank test). **d** Live-cell imaging of mCherry-BMAL1-expressing GFP-G3BP1 KI cells exposed to 20 μM of sodium arsenite (see Movie S3, 4). **e** The cell death ratio of mCherry- and stress granule-positive cell, ***P ≤ 0.0001 by log-rank test. **f**, **g** Caspase-3 activation in GFP-G3BP1 KI cells transfected with *Bmal1* siRNA (**f**) or mCherry-BMAL1 expression plasmid (**g**). At 72 h after siRNA transfection, cells were treated with 50 μM SA for 12 h before analysis. For cells with mCherry-BMAL1 overexpression, they were treated 48 h post transfection with 20 μM SA for 7 h before analysis. Representative images of activated caspase-3 staining and the quantification of percentage of cell death as indicated by activated caspase-3 are shown for each condition (mean ± S.E.M.; *n* = 3 independent experiments, with 10 fields scored per experiment, each field >100 cells, ***P ≤ 0.001 by unpaired Student’s-*t*-test. Scale bar = 20 μm)

As stress granule formation is a pro-survival response during stress^10^, we evaluated the relationship of stress granule formation with cell death under stress when circadian cues are altered. We first used specific small interfering RNA (siRNA) to silence BMAL1 expression, and then treated GFP-G3BP1 KI cells with 20 μM sodium arsenite and recorded cell morphology and the appearance of stress granules using live imaging (fluorescence and bright field) for up to 600 min (Fig. 4a). The timing for stress granule appearance and the fate of these stress granule-positive cells were analyzed individually. In cells with silenced BMAL1, there was a delayed appearance of stress granules, but higher percentage of cells with stress granules (Fig. 4b), consistent with the patterns seen in cells exposed to transient stress shock (Fig. 3a). Interestingly, these stress granule-positive, BMAL1-silenced cells exhibited significant resistance to stress-induced cell death (Fig. 4c). In contrast, live imaging analysis of cells overexpressing mCherry-BMAL1 showed increased sensitivity to stress-induced cell death (Fig. 4d, e). To validate the effect of stress-induced cell death by altered BMAL1 expression, we examined activated caspase-3, an indicator for apoptotic cell death, in control or BMAL1-silenced cells, or in cells overexpressing BMAL1. While BMAL1 knockdown protected cells against stress-induced cell death (Fig. 4f), BMAL1 overexpression potentiated it (Fig. 4g). Therefore, transient low expression of BMAL1 facilitated the formation of stress granules, and protected cells against stress-induced cell death.

**Fig. 5:**
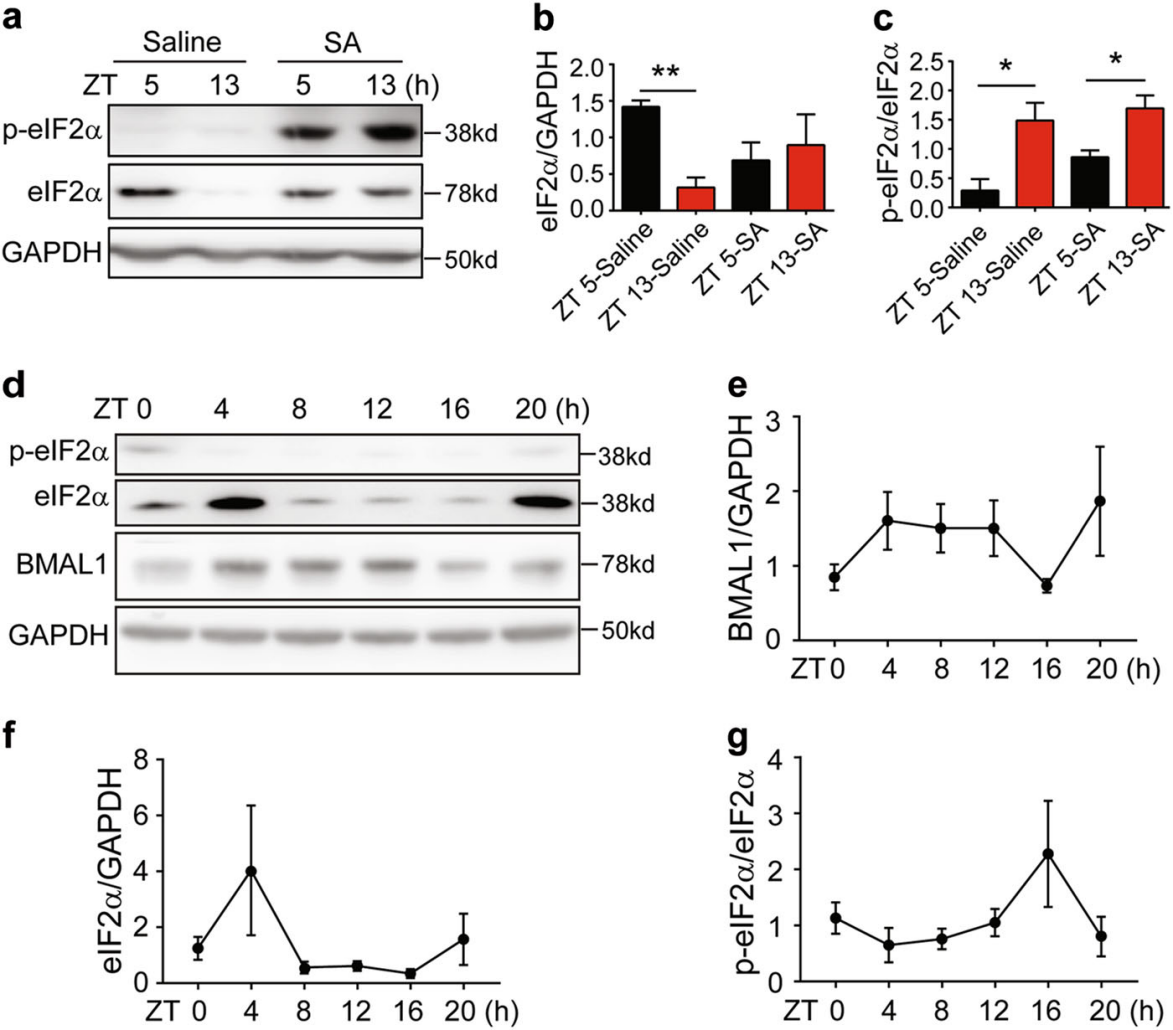
Circadian oscillation of eIF2α and stress granules in mouse liver. **a**–**c** Protein expression of eIF2α and p-eIF2α in the liver tissues of the wild-type mice at zeitgeber time (ZT) 5 and ZT 13. Mice were treated with saline or sodium arsenite (10 mg/kg) for 1 h before tissue harvest. Values in (**b**, **c**) represented mean ± S.E.M.; *n* = 3 independent experiments, *P ≤ 0.05, **P ≤ 0.01 by unpaired Student’s-*t*-test. **d**–**g** Protein levels of BMAL1, eIF2α, and p-eIF2α at indicated zeitgeber times. **f** Representative immunoblots showing the expression levels of eIF2α, p-eIF2α, BMAL1, and GAPDH. **e**–**g** Quantification of relative expression of indicated proteins (mean ± S.E.M.; *N* = 5 mice per time point)

To investigate the mechanism that may account for the regulation of stress granules by circadian proteins, we examined whether it could be related to eIF2α, as eIF2α phosphorylation is a key signal to initiate the stress granule assembly^12^. We first examined the total and phosphorylated eIF2α in mouse liver collected at ZT 5 and ZT 13, with or without sodium arsenite exposure. At ZT 13, there was a significant drop of total eIF2α level at both the protein and mRNA levels (Fig. 5a, b, Supplementary Fig.4a), but the absolute phosphorylated eIF2α level remained stable or slightly increased (Fig. 5a). When exposed to arsenite stress, the mouse liver showed a clear increase of total and phosphorylated eIF2α (Fig. 5a). Interestingly, the ratio of phosphorylated eIF2α to total eIF2α was significantly elevated at ZT 13, even in the absence of stress insults (Fig. 5c), which may account for the higher basal stress granule levels at ZT 13 (Fig. 1a, b).

The significant difference of total eIF2α also suggested a possibility that eIF2α could be circadian regulated. We analyzed published proteomic and transcriptome dataset from mouse liver^52^ and found that the expression of eIF2α was indeed oscillating (Supplementary Fig. 4b, c), and the oscillation would be shifted in CRY1/2 KO mice^53^ or weakened in BMAL1 KO mice^54^ (Supplementary Fig. 4d, e). In addition, eIF2α expression also oscillates in baboons, although with a peak around ZT 12–16 in the brain (Supplementary Fig. 4f, g)^55^. To thoroughly examine the change of eIF2α at various circadian time, we collected liver and brain tissues from entrained mice every 4 h (*n* = 5 mice at each time point) and determined the expression of eIF2α and BMAL1 (Fig. 5d) as well as the spontaneous stress granule formation at each time point (Supplementary Fig. 5a, b). The BMAL1 protein showed oscillating expression as expected, with relatively lower expression at ZT 16–20 (Fig. 5e), which also coincided with increased abundance of stress granules (Supplementary Fig. 5a, b). Consistent with our analysis of proteomic and transcriptome data (Supplementary Fig. 4b–g), the oscillation of total  eIF2α was observed in liver (Fig. 5d, f), cortex, and hypothalamus (Supplementary Fig. 5c, d). Remarkably, the relative expression of phosphorylated eIF2α in the liver also showed an oscillating pattern, but in the opposite phase as the total eIF2α (Fig. 5g). It is worth noting that the absolute level of phosphorylated eIF2α did not vary significantly between ZT 4 and ZT 16 (Fig. 5d), even though the total eIF2α expression was at peak and trough, respectively. Therefore, at ZT 12–16 when the total eIF2α was at trough, even minor phosphorylation of eIF2α would quickly reduce the pool of unphosphorylated eIF2α, increase the ratio between phosphorylated and total eIF2α, and cause translation arrest and stress granule formation.

**Fig. 6:**
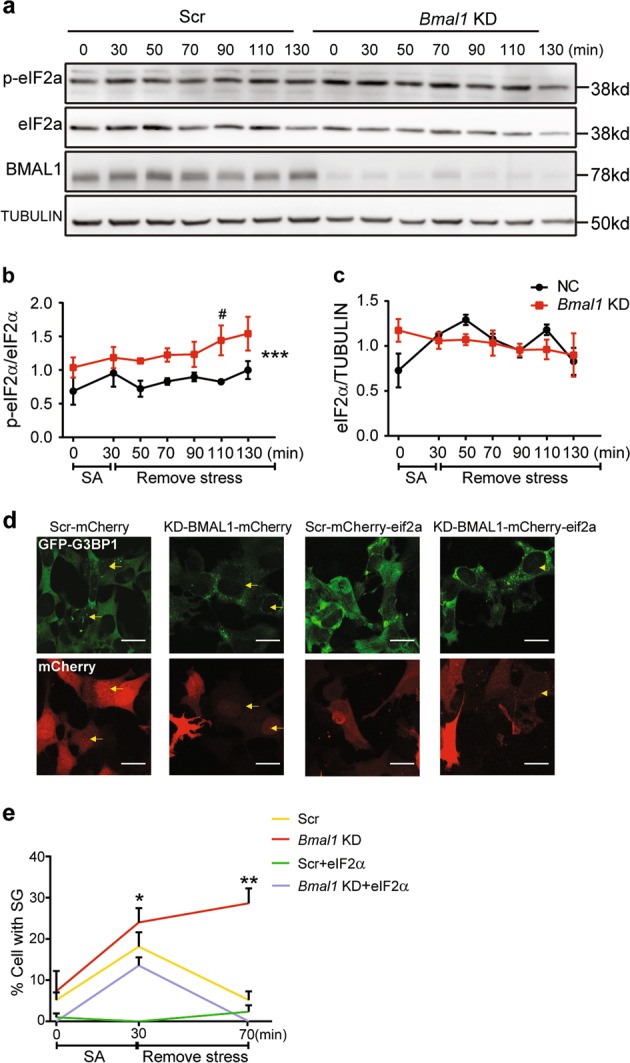
Reduced  BMAL1 expression increased the ratio of P-eIF2α to total eIF2α and the number of stress granules. **a**–**c** Protein levels of p-eIF2α, eIF2α, and BMAL1 at indicated time points after stress shock in cells with reduced BMAL1 expression. Representative immunoblots are shown in **a**, and the quantification of p-eIF2α and eIF2α are presented in **b** and **c**, respectively (mean ± S.E.M.; *n* = 3 independent experiments, two-way analysis of variance (ANOVA) with Sidak’s multiple comparison test, * represents the *P* value of two-way ANOVA among 50–130 min ***P ≤ 0.001; ^#^ represents the *P* value of Sidak’s multiple comparison, ^#^*P* ≤ 0.05). **d**, **e** Expression of total eIF2α blocked the increased stress granule formation by BMAL1 silencing. mCherry-eIF2α or mCherry was transfected in GFP-G3BP1 knock-in (KI) cells with control or small interfering RNA (siRNA) targeting BMAL1, and the number of stress granules formed was analyzed after stress shock. Representative images are shown in **d**, and the quantification is presented in **e** (mean ± S.E.M.; *n* = 150–200 cells per sample, *P ≤ 0.05, **P ≤ 0.01 by unpaired Student’s *t*-test between KD-BMAL1 and KD-BMAL1-eIF2α. Scale bar = 20 μm). Yellow arrows indicate mCherry and SG double-positive cells

To directly test whether reduced pool of unphosphorylated eIF2α could contribute to increased stress granule formation in cells with low expression of BMAL1, we first transiently silenced the BMAL1 expression using siRNA, and then stress shocked SH-SY5Y cells with sodium arsenite. While the total eIF2α slightly decreased, the phosphorylated eIF2α clearly increased (Fig. 6a–c), suggesting reduced abundance of unphosphorylated eIF2α. We then transiently transfected mCherry-tagged eIF2α in GFP-G3BP1 KI cells pretreated with BMAL1 or control siRNA, and examined the stress granule formation after stress shock by immunofluorescence microscopy. eIF2α expression significantly suppressed the formation of stress granules (Fig. 6d, e). Therefore, decreased pool of unphosphorylated eIF2α in cells with low BMAL1 expression contributed to the formation of stress granules.

## Discussion

In this study, we have provided evidence to show that stress granule formation and dynamics are affected by circadian cues, and this regulation is caused by oscillating eIF2α expression. Furthermore, we have discovered that altering the circadian gene expression by decreasing the expression of BMAL1 could promote stress granule formation and protect arsenite stress-induced apoptosis. Therefore, our findings have shed new light on the circadian influence on the cellular defense mechanisms against stress insults and could help us better understand the circadian influence on neurodegeneration and cancer.

The regulation of stress granule formation by phosphorylation of the translation initiation protein eIF2α is well documented^56^; however, the diurnal oscillation of eIF2α has never been reported. Stress granules are quickly assembled when cells are exposed to sudden increase of environmental and cellular stress^57^. In contrast, the rhythmic changes in physiology and behaviors governed by circadian clock usually happen at much slower pace. The oscillating eIF2α expression happens to seamlessly connect the circadian rhythm and stress granule formation. We have found that in mouse liver the eIF2α expression troughs when the BMAL1 expression is at nadir at early night time, also an active phase for nocturnal animals with increased lipid peroxidation^46,58^. Phosphorylation of eIF2α under stress condition at night would act as a fast-acting signaling event to further reduce the small pool of unphosphorylated eIF2α that is required for translational initiation, thus causing translation arrest and stress granule formation. On the other hand, the same stress insult at day time (ZT 5) in mouse would cause a much smaller impact on stress granule formation. This regulation provides an efficient cellular mechanism to manage increased stress during active phase for nocturnal animals. Interestingly, the peak expression of BMAL1 and eIF2α in primates are between ZT 12 and 16, about anti-phase to that in mice^55^ (Supplementary Fig. 4f, g). Therefore, the regulation of stress granules by oscillating eIF2α could be a conserved mechanism in mammals to cope with increased stress during active phase.

Although eIF2α oscillation and phosphorylation appear to be the main driver of circadian control of stress granules, other mechanisms may also contribute to the circadian regulation of stress granules. Based on our analysis of stress granule proteome^13^ and circadian-regulated transcriptome^59^, the expression of a score of stress granule component proteins, such as FUS, TDP43, and CRIBP, are under circadian control. Given that the stress granule assembly and dynamics are heavily influenced by protein-protein interaction^14^, the abundance of these proteins at various circadian time may also affect stress granules. In addition, the stress granule assembly and dynamics are affected by the presence of various ATPase, such as valosin-containing protein (VCP), in the granules^14,60^ and depend on the adenosine triphosphate (ATP)^13,14^. Given that ATP production could also be affected by circadian regulation of mitochondrial biogenesis^61,62^, it would be of interest to evaluate whether ATP abundance may contribute to the timing and sensitivity of stress granule formation.

One surprising finding from our study is the protective effect of low BMAL1 expression during stress response. BMAL1 KO mice have significant disrupted circadian rhythm and metabolism^63,64^ and have shortened life span with early aging pathologies^65^. In another study, BMAL1 KO mice show synaptic degeneration and increased neuronal oxidative damage^66^. However, transiently reduced BMAL1 expression promotes the stress granule formation and was protective in our study. Our observations are consistent with a recent paper showing the anti-apoptotic effect of reduced BMAL1 expression during unfolded protein response^67^. We have further demonstrated the difference in the stress response between cells with BMAL1 knock-out background and transient reduction (Supplementary Fig. 3a, b; Fig. 3a, c). Therefore, unlike the harmful effects of long-lasting BMAL1 depletion, daily oscillation of BMAL1 and eIF2α could increase the sensitivity to stress insults during the active dark phase in mice and could transiently stop protein translation by forming protective stress granules. It is not surprising for multifunctional proteins such as BMAL1 to exhibit divergent effects on cell survival and death based on the duration and abundance of their expression. For example, transient induction of the wild-type p53 is pro-survival while accumulation of p53 leads to apoptosis^68^. It will be of interest to examine the stress granule formation in detail in BMAL1 KO mice during the aging process to fully assess the role of BMAL1 in stress response and cell death.

As circadian rhythm is known to decay with aging, our study may provide a new cellular mechanism to account for the abnormal stress granule assembly, disassembly, and dynamics that could contribute to increased risks for cancer, viral infection, and neurodegenerative diseases in aged population. Remarkably, a group of proteins implicated in the pathogenesis of ALS and/or FTD are components of stress granules and could even participate in the assembly or disassembly of stress granules^18,28,60,69,70^, thus further strengthening the link between stress granule formation and neurodegenerative diseases. Interestingly, some mutations that cause neurodegenerative diseases could lead to altered circadian regulation by causing neuronal loss in brain regions affecting circadian and sleep^71,72^, or directly participating in circadian regulation. With time, they could start a feedforward cycle to affect stress response and accelerate the development of neurodegenerative diseases. Consistent with this idea, our recent studies have shown that ALS/FTD-associated FUS is a circadian regulator^73^ and that F521C KI rats exhibit circadian abnormalities as early symptoms^42^.

Chronotherapy has been practiced to improve the efficacy of anti-cancer drugs by modulating their pharmacokinetics and pharmacodynamics^45,74^. DNA repair and apoptosis, which some anti-cancer drugs target, are circadian regulated^43,74^. Together with these pathways, stress granules could be part of the circadian-regulated components contributing to the molecular cellular mechanisms in chronotherapy in cancer. Stress granule formation promotes cancer survival and resistance to chemotherapy agents^3^. Mutant Kras enhances cancer cell survival by stimulating stress granules^75^. Conversely, suppressing stress granules could increase the drug sensitivity in Hela cells^76^. Consistent with our findings showing increased stress granule formation in the evening, a recent study has reported that chemotherapy agent cisplatin causes less damage and side effects in the wild-type mice when the drug was delivered at that time^77^. Therefore, our current study will prompt the future investigation to decipher the circadian regulation of stress granules in cancer cells and help to optimize chronotherapy regimen to maximize the drug efficacy in cancer cells while minimizing the side effects in normal cells by considering the timing of stress granule formation.

## Methods

### Animals

All animal works were performed in accordance with the regulations by the Animal Care and Use Committee of Institute of Neuroscience, Chinese Academy of Science. Mice were killed under anesthesia, and all efforts were made to minimize mice suffering.

The 7–8-week-old wild-type C57BL/6J male mice were purchased from SLAC (Shanghai Laboratory Animal Center) and were maintained on a 12 h light:12 h dark (lights on at 7 a.m.) throughout the study. Mice were housed in the facility for 2 weeks before experiments. Food and water were available ad libitum. At ZT 4 (zeitgeber time 4, means 4 h after lights on) and ZT 12, the mice received intraperitoneal injection of saline or sodium arsenite (10 mg/kg) and the liver tissues were harvested at ZT 5 and ZT 13 for immunofluorescence and western blot. For the experiment to evaluate eIF2α circadian oscillation and stress granule formation oscillation, mice were single-housed for at least 2 weeks prior to experimental use, and 5 mice were harvested for small part of liver, cortex, and hypothalamus at each time point every 4 h (starting at zeitgeber time 0) for western blot. The mouse livers were then perfused through hepatic portal vein with 4% paraformaldehyde for subsequent immunofluorescence labeling.

### Antibodies and drugs

The primary antibodies used are: mouse anti-eIF2α (sc-133132 Santa Cruz Biotechnology, 1:1000 for western blot); rabbit anti-phospho-eIF2α (9721 Cell Signaling Technology, 1:1000 for western blot); rabbit anti-BMAL1(14020 Cell Signaling Technology, 1:1000 for western blot); mouse anti-GAPDH (60004 Proteintech, 1:1000 for western blot); mouse anti-β-tubulin (M20005, Abmart; 1:3000 for western blot); rabbit anti-PABP1 (ab2060, Abcam, 1:100 for immunofluorescence), rabbit anti-YB1 (ab76149, Abcam, 1:200 for immunofluorescence); and rabbit anti-cleaved-caspase-3 (9661 Cell Signaling Technology, 1:200 for immunofluorescence). The drug used was sodium arsenite (S7400-100G, Sigma, 100 mg/kg for mouse and 20 or 50 μM for cells).

### Plasmids and siRNA

The plasmids, mCherry-tagged BMAL1 and eIF2α, were generated by PCR using human complementary DNA (cDNA) library and cloned into the EGFP-C1 vector (Invitrogen), with EGFP changed to mCherry.

The siRNA constructs targeting the human *BMAL1* were synthesized at Genepharma with the following sequences:

BMAL1-996 (sense, 5’-CCUCAACUACAGCCAGAAUTT-3’; antisense, 5’-AUUCUGGCUGUAGUUGAGGTT-3’);

BMAL1-1565 (sense, 5’-GCACAUCGUGUUAUGAAUATT-3’; antisense, 5’-UAUUCAUAACACGAUGUGCTT-3’);

BMAL1-12247 (sense, 5’-GCCUUCAGUAAAGGUUGAATT-3’; antisense, 5’-UUCAACCUUUACUGAAGGCTT-3’).

Cy3-labeled BMAL1 siRNA (sense, 5’-GCACAUCGUGUUAUGAAUATT-3’; antisense, 5’-UAUUCAUAACACGAUGUGCTT-3’);

Cy3-labeled Scramble siRNA (sense, 5’-UUCUCCGAACGUGUCACGUTT-3’; antisense, 5’-ACGUGACACGUUCGGAGAATT-3’);

Scramble siRNA (sense, 5’-UUCUCCGAACGUGUCACGUTT-3’; antisense, 5’-ACGUGACACGUUCGGAGAATT-3’).

For *Bmal1* siRNA transfection, total of 5 μL of above constructs were mixed at 1:1:1 ratio for cells cultured in 3.5 cm dishes.

### Western blot

The proteins from mouse tissues or cultured cells protein were extracted with RIPA lysis buffer (150 mM NaCl, 50 mM Tris (pH = 8.0), 1% NP40, 1% sodium deoxycholate, 0.1% SDS) supplemented with protease inhibitor cocktail (Roche) and phosphatase inhibitor cocktail (Roche) as needed. Proteins were resolved by sodium dodecyl sulfate–polyacrylamide gel electrophoresis (SDS-PAGE), and the protein bands were visualized using Bio-Rad western ECL substrate kit. The band intensity in immunoblots was determined by Bio-Rad Quantity One software.

### Cell culture and transfection

MEFs were collected from E13.5 embryos using pregnant mice from *Nr1d1* KO mice or *BMAL1* KO mice mating pairs. E13.5 embryos were eviscerated. Embryos’ epidermis was cut into piece then digested into single cell with Tyrisin (Tyrisin-EDTA 0.25% phenol red Thermo Fisher). Large cell lumps were removed through cell strainer (40 μm nylon FALCON).

MEFs and SH-SY5Y cells were cultured at 37 °C in 5% CO_2_ in Dulbecco’s modified Eagle’s medium (Invitrogen), supplemented with 10% fetal bovine serum (Invitrogen) and antibiotics (penicillin and streptomycin, HyClone, SV30010). Cells were transfected as needed using Lipofectamine 2000 reagent (Invitrogen) or Lipofectamine RNA-MAX (Invitrogen). The total amount of plasmid DNA or siRNA was adjusted to 5 μg per 3.5 mm dish. Cells were harvested at 48 or 72 h (siRNA) post transfection for western blotting.

### Quantitative reverse transcriptase PCR

For real-time PCR, total RNAs were extracted from cells using Trizol. The full-length cDNA library was constructed by reverse transcription PCR using PrimeScript^TM^ RT Master Mix Perfect Realtime (RR036A, Takara). Quantitative reverse transcriptase PCR was performed using iQ^TM^ SYBR@ Green Supermix (1708882, Bio-Rad) with Bio-Rad CFX Connect real-time PCR system.

### Generation of GFP-G3BP1 KI SH-SY5Y cell line by CRISPR/cas9

The genomic sequences surrounding the coding region of human G3BP1 N-terminal (±200 bp) were analyzed for potential CRISPR/Cas9 cleavage sites, and the CRISPR design tool (http://crispr.mit.edu/) was used to design single-guide RNA (sgRNA). The five sgRNAs with high predicted score were synthesized and subcloned into the pX330 vector (Addgene) containing the flanking sgRNA sequences and a codon-optimized Cas9.

For selecting the sgRNA leading to optimal Cas9 cleavage, sgRNA plasmid which also expresses mCherry was transfected in human neuroblastoma cell line SH-SY5Y (ATCC). After 48 h, transfected cells were dissociated with trypsin and sorted using flow cytometry to identify mCherry-positive cells. T7 endonuclease I digestion patterns of PCR-amplified genomic DNA from these cells were analyzed to determine the optimal sgRNA that can lead to highest cleavage efficiency. Donor sequences containing GFP and 20 bp flanking G3BP1 sequences were cloned to donor vector (pX85, Addgene). To facilitate efficient homologous recombination, the selected sgRNA target sequences were fused to the G3BP1 homologous arms as previously described^78^ At 24 h after transfection of donor and sgRNA plasmids (1:1), the SH-SY5Y cells were selected for mCherry-positive cells using flow cytometry. After 1–2 weeks, GFP-positive cells were sorted and selected for expansion and functional validation using immunocytochemistry.

### Fluorescence recovery after photobleaching

GFP-G3BP1 KI cells were plated in 25 mm glass dish for 24 h. Cells were then transfected with plasmids or siRNAs. After 48 or 72 h of incubation, cells were treated with sodium arsenite for 30 min to induce stress granules. The stress granules in siRNA-positive (CY3) or mCherry-positive cells were  photobleached and GFP intensity was measured before and after bleaching. For FRAP experiments in MEF cells, WT and *Nr1d1* KO or *BMAL1* KO MEF cells were transfected with GFP-G3BP1 plasmids. After 48 or 72 h of incubation, cells were treated with sodium arsenite to induce stress granules. Images were taken with an inverted laser scanning confocal microscope (Nikon A1R) with 60× TIRF oil immersion lens equipped with a humidified 5% CO_2_ incubator at 37 °C with an environment-controlled chamber.

### Immunofluorescence and imaging

SH-SY5Y cells were cultured on glass coverslips. Cells were fixed with 4% paraformaldehyde for 30 min, permeabilized with 0.25% Triton X-100 for 10 min, blocked with 3% bovine serum albumin/phosphate-buffered saline (PBS) (Blocking buffer) for 30 min. The cells were then incubated with specific primary antibodies for 12 h at 4 °C, followed by incubation with appropriate Alexa Fluor-conjugated secondary antibodies (Donkey anti-Mouse/Rabbit/Goat-488/546/647, Invitrogen; 1:500) for 2 h at room temperature and mounted with a drop of Vectashield mounting solution (94010, Vector Laboratories, Inc.).

Mouse liver was perfused and fixed with 4% paraformaldehyde for 24 h and then transferred to 30% sucrose for 48 h. At least 3 frozen sections (8 μm) of each tissue were mounted on glass slide. After permeabilization with 0.25% Triton X-100/PBS for 1h, slides were soaked into 95 °C antigen retrieval solution (10 mM sodium citrate, 0.05% Tween 20, pH = 6.0) for 20 min and then transferred into water for 10 min at room temperature, followed by blocking with Blocking buffer for 30 min. To detect the formation of stress granules in all cell types in the liver tissues, frozen sections were stained with anti-PABP1 or YB1 antibody, and fluorescence-conjugated secondary antibodies. We chose >0.5 μm cut-off as a more stringent standard to identify large stress granules. Stress granule diameter was measured using Fiji software. Images were taken with an inverted laser scanning confocal microscope (Nikon A1R) with 60× TIRF oil immersion lens.

### Live-cell imaging and hazard analysis

Live-cell imaging experiments were performed on Olympus FV10I use 60× water objective lens. GFP-G3BP1 KI cells were plated in 29 mm dish with glass bottom. Before imaging, the medium was changed to FluoroBrite medium containing sodium arsenite. During imaging, cells were maintained in a humidified 5% CO_2_ incubator at 37 °C with an environment-controlled chamber. Multi-positions and *Z*-stacking were used during the imaging. During imaging, 488 and 546 nm laser was used with laser intensity set below 10% to avoid phototoxicity. Time-lapse images were collected at 6 min interval for 400–600 min and the images were analyzed with Fiji software.

### Statistical analyses

For data analysis, the results were presented as mean ± S.E.M., with statistical significance analyzed using Student’s *t*-test in all the experiments. Two-way analysis of variance (ANOVA) was used for analyzing stress granule formation with time in mouse liver, live-cell imaging, eif2α, and phosphorylated eIF2α level in sodium arsenite-treated SH-SY-5Y cells (*P ≤ 0.05; **P ≤ 0.01; ***P ≤ 0.001; GraphPad, Prism 5).

## Supplementary information

supplementary figure legends

Supplementary Fig. 1

Supplementary Fig. 2

Supplementary Fig. 3

Supplementary Fig. 4

Supplementary Fig. 5

Legends for videos

Supplementary movie. 1

Supplementary movie. 2

Supplementary movie. 3

Supplementary movie. 4
