# Supplementary material for: Circadian control of stress granules by oscillating EIF2α
Source: Cell Death Dis. 2019 Mar 4;10(3):215. doi: 10.1038/s41419-019-1471-y (PMC6399301; doi:10.1038/s41419-019-1471-y)
Supplement: Supplementary file 1 — supplementary figure legends [file 41419_2019_1471_MOESM1_ESM.docx]

**Fig. S1. Stress granules in mouse liver at ZT 5 and ZT 13**

(a) BMAL1 protein level at ZT 5 and ZT 13. (b) The presence of stress granules in liver cells was validated with another stress granule marker YB-1, which labels stress granule and processing body under normal condition but can label stress granule under stress conditions such as sodium arsenite exposure. (c) Quantification of YB-1 positive stress granules at ZT 5 and ZT 13. (Mean ± S.E.M.; n = 3 mice per group with 800-950 cells examined per mouse, Two-way ANOVA with Tukey’s multiple comparison **P* ≤0.05, scale bar = 10 μm)

**Fig. S2. Generation of GFP-G3BP1 knock-in cell line**

**(a) Schematic representation of** sgRNA sequences and the targeting site **at human** G3BP1**locus.** (b) Analysis of the cleavage efficiency of five sgRNAs. Yellow arrow indicates the selected sgRNA used to generate GFP-G3BP1 knock-in cell line. (c-d) (c) Western bolting showing the protein expression of BMAL1 in synchronized GFP-G3BP1 knock-in cell line. Cells were treated with 100nM Dexamethasone for 1 h when cells reach confluence, then the cells were cultured in the low serum condition (DMEM with 1% FBS) for 36 h followed by harvesting every 6h. (d) The quantification was shown on the left (mean ± S.E.M.; N=3 independent expreriments.)

**Fig. S3. Stress granule dynamics and formation in BMAL1 KO or NR1D1 KO MEF cells**

(a) WT and BMAL1-KO MEF cells were transfected with GFP-G3BP1 and subsequently treated with 50 μM SA for 30 min. followed by FRAP analysis (b) Representative images show the SG formation in WT or BMAL1-KO cells treated with 50 μM SA for 30 min. The percentage of cells with stress granules under indicated condition was quantified (mean ± S.E.M.; n = 2 independent experiments, with 20 fields for each time point, at least 50 cells per field. by unpaired Student’s-*t*-test, scale bar = 20 μm). (c-d) WT and NR1D1-KO MEF cells were transfected with GFP-G3BP1 and subsequently treated with 50 μM SA for 30 min. followed by FRAP analysis (mean ± S.E.M.; n = 9-10; **P* ≤0.05, ****P* ≤ 0.001 by unpaired Student’s-*t*-test). (d) Mobile fraction calculated from the FRAP analysis

**Fig. S4. Circadian oscillation of eIF2α protein and mRNA in the mouse liver**

(a) Real-time PCR results showing *eIF2α* mRNA level in mouse liver treated with sodium arsenite (10 mg/kg) for 1 h at ZT 5 and ZT 13. Values were normalized to control group as relative expression with respect to endogenous control gene GAPDH (mean ± S.E.M.; n = 3; **P* ≤ 0.05). (b-c) Analysis of the circadian expression of eIF2α protein (b) and mRNA (c) using published proteomic and RNA-seq data in mouse liver data ^52^. (d) Analysis showing altered eIF2α protein oscillation pattern in *Cry1/2* KO mouse ^53^. (e) Altered *eIF2α* mRNA oscillation patterns in the liver of *Bmal1* KO mice ^54^. (f), (g) *eIF2α* mRNA oscillation patterns in the SCN (suprachiasmatic nucleus) and SN (substantia nigra) of baboon^55^

**Fig. S5. Stress granules formation oscillation in mouse liver and oscillated eIF2α protein expression in mouse cortex and hypothalamus**

(a-b) Immunofluorescence microscopy showing the stress granule formation in the liver of free-running wild-type mice (ZT: zeitgeber time), with quantification shown in panel (b) (Mean ± S.E.M.; N = 4 mice were sacrificed at each time point, 350-500 cells examined per mouse scale bar=10 μm). (c-d) Protein levels of BMAL1 and eIF2α in the cortex (c) and hypothalamus (d) were analyzed at indicated zeitgeber times. (n = 5 mice per time point)
