# Supplementary figures and images for: Circadian control of stress granules by oscillating EIF2α
Source: Cell Death Dis. 2019 Mar 4;10(3):215. doi: 10.1038/s41419-019-1471-y (PMC6399301; doi:10.1038/s41419-019-1471-y)

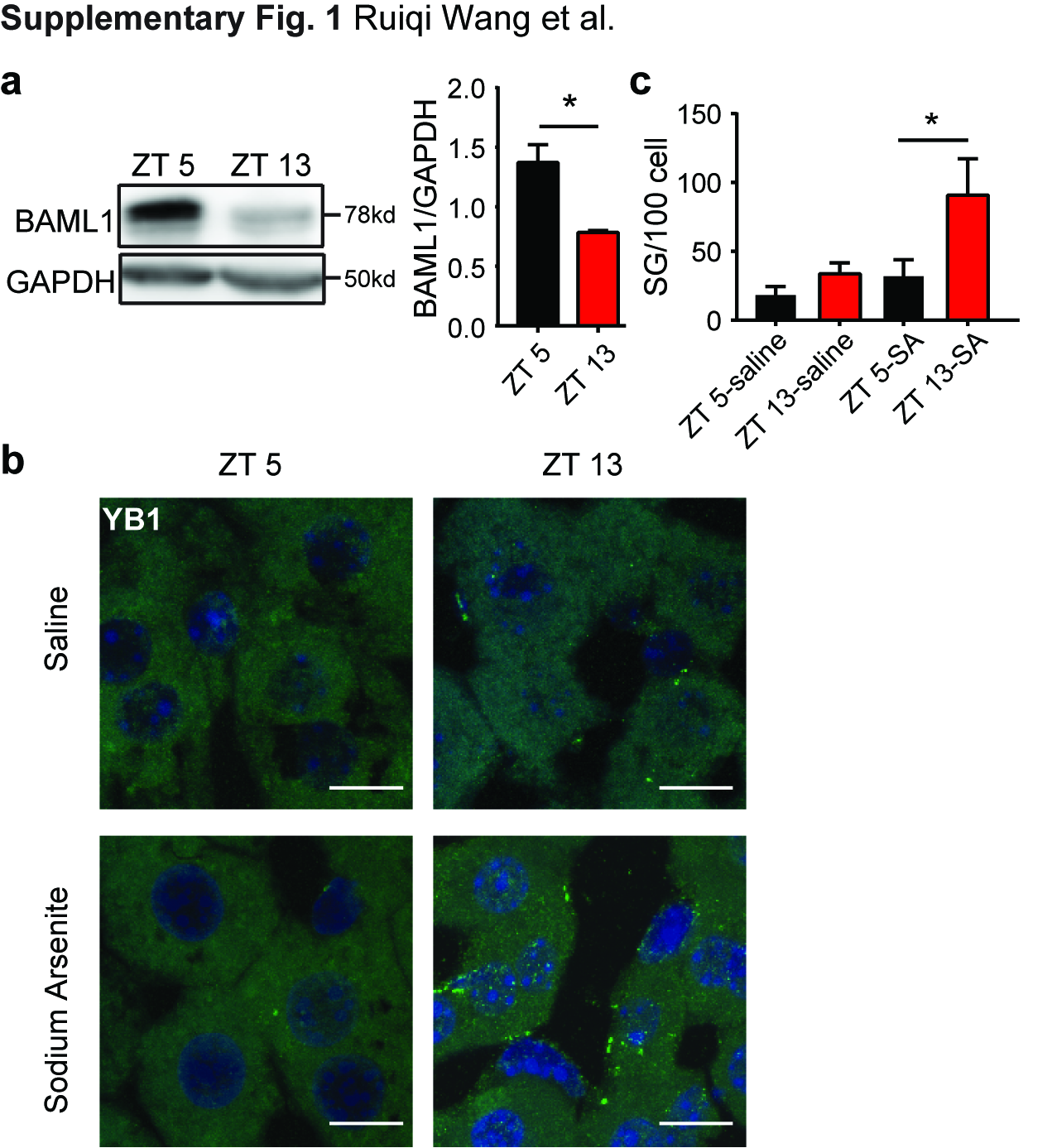

Supplement: Supplementary file 2 — Supplementary Fig. 1 [file 41419_2019_1471_MOESM2_ESM.tif]

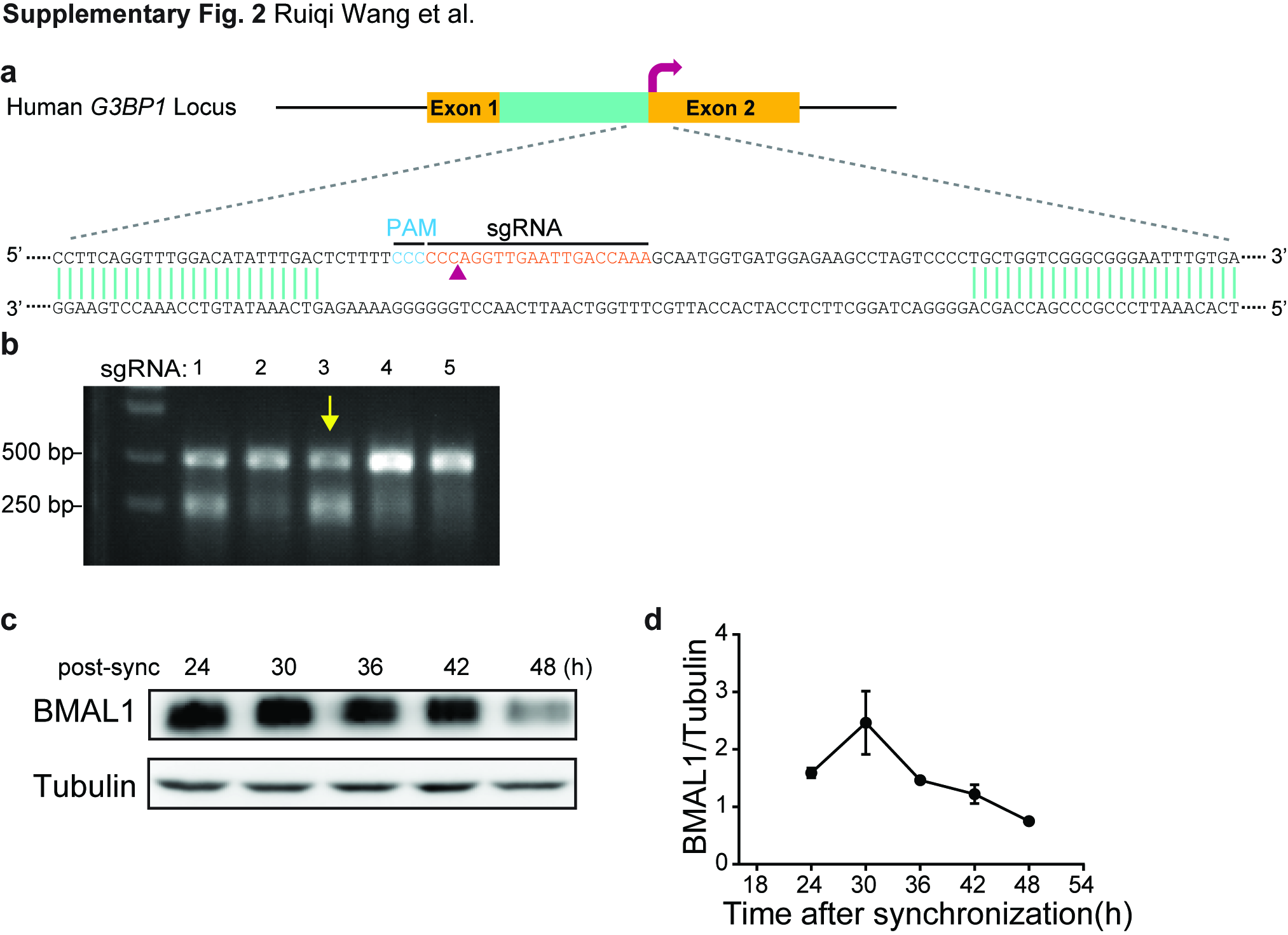

Supplement: Supplementary file 3 — Supplementary Fig. 2 [file 41419_2019_1471_MOESM3_ESM.tif]

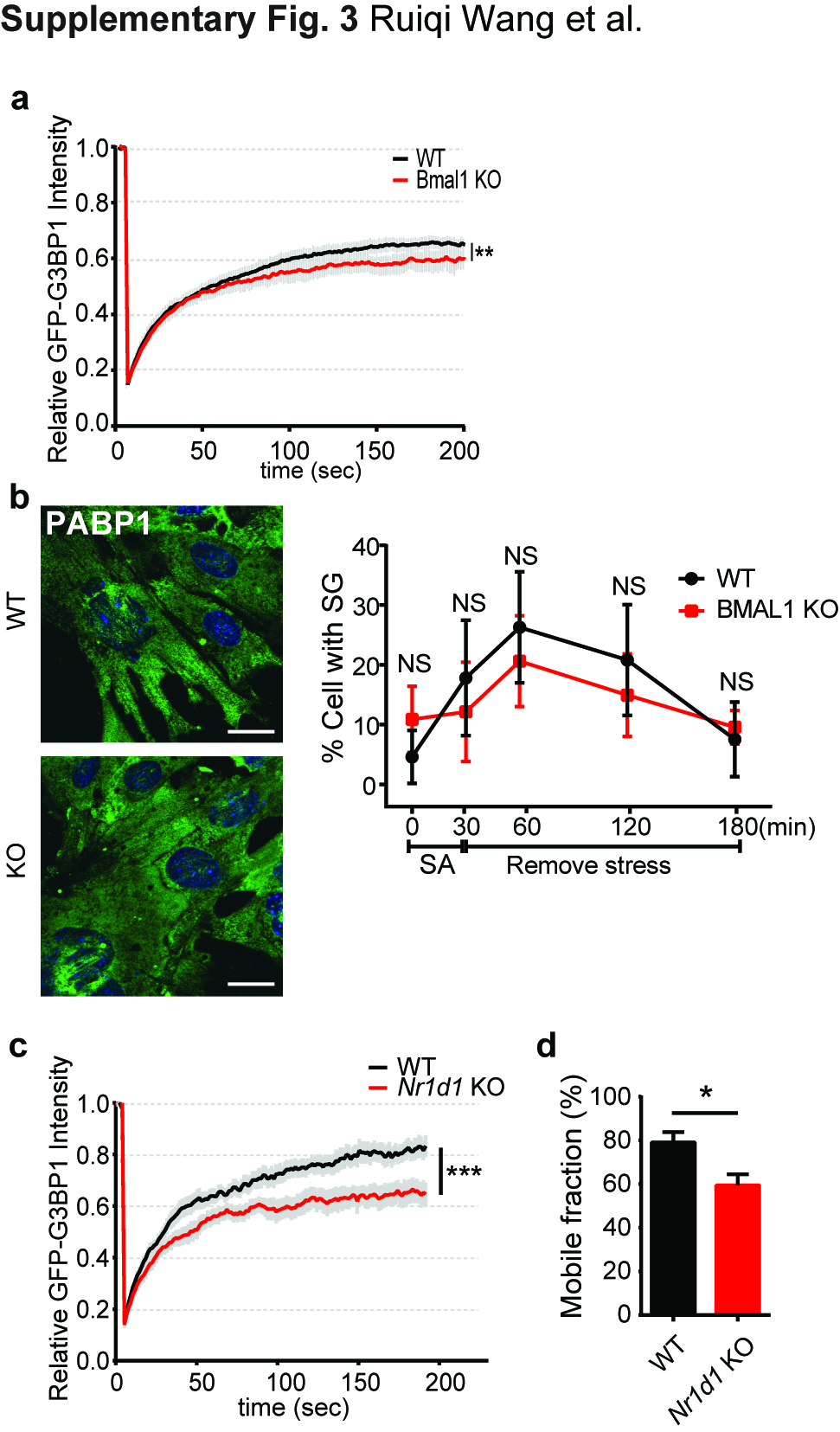

Supplement: Supplementary file 4 — Supplementary Fig. 3 [file 41419_2019_1471_MOESM4_ESM.tif]

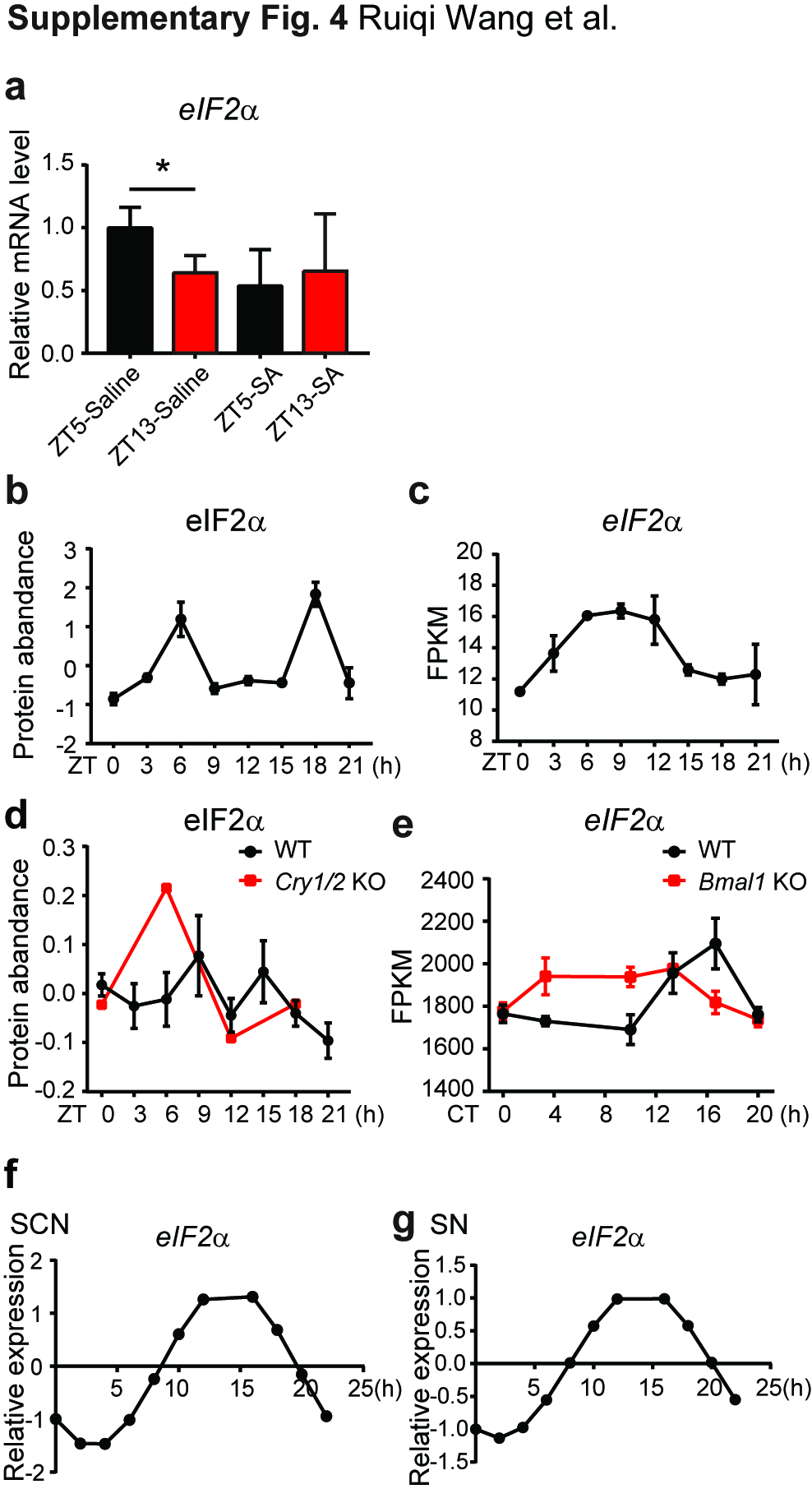

Supplement: Supplementary file 5 — Supplementary Fig. 4 [file 41419_2019_1471_MOESM5_ESM.tif]

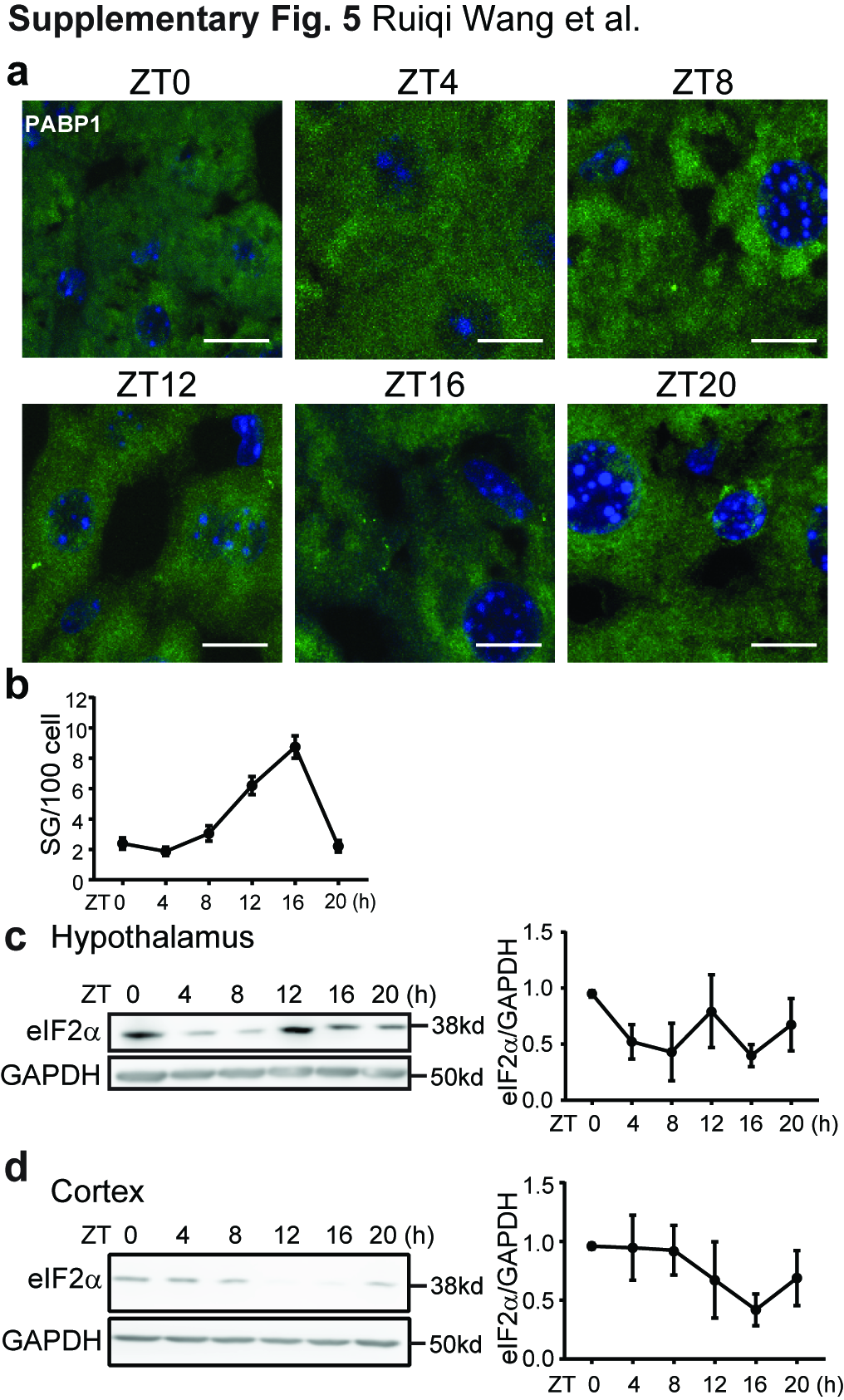

Supplement: Supplementary file 6 — Supplementary Fig. 5 [file 41419_2019_1471_MOESM6_ESM.tif]
