# Supplementary material for: Circadian control of stress granules by oscillating EIF2α
Source: Cell Death Dis. 2019 Mar 4;10(3):215. doi: 10.1038/s41419-019-1471-y (PMC6399301; doi:10.1038/s41419-019-1471-y)
Supplement: Supplementary file 7 — Legends for videos [file 41419_2019_1471_MOESM7_ESM.docx]

**Legends for videos**

**Movie S1. Live Cell Imaging of SG formation and cell death in GFP-G3BP1 KI** **cells transfected with scrambled siRNA (refer to Fig. 4A)**

Cells were exposed to 20 μM of sodium arsenite for 600 min with multiple fields recorded every 2 minutes. A representative field is shown here, with yellow arrow indicating a representative SG positive cell analyzed. Red arrow indicates the timing this SG positive cell starting to show apoptotic morphology.

**Movie S2. Live Cell Imaging of SG formation and cell death in GFP-G3BP1 KI** **cells transfected with *Bmal1* siRNA (refer to Fig. 4A)**

**Movie S3. Live Cell Imaging of SG formation and cell death in GFP-G3BP1 KI** **cells transfected with mCherry plasmid (refer to Fig. 4D)**

Cells were exposed to 20 μM of sodium arsenite for 400 min. A representative field is shown here, with yellow arrow indicating a representative cell analyzed. Red arrow indicates the timing this cell starting to show apoptotic morphology

**Movie S4. Live Cell Imaging of SG formation and cell death in GFP-G3BP1 KI** **cells transfected with mCherry-BMAL1 (refer to Fig. 4D)**
